# A phase I study of AST1306, a novel irreversible EGFR and HER2 kinase inhibitor, in patients with advanced solid tumors

**DOI:** 10.1186/1756-8722-7-22

**Published:** 2014-03-11

**Authors:** Jian Zhang, Junning Cao, Jin Li, Yifan Zhang, Zhiyu Chen, Wei Peng, Si Sun, Naiqing Zhao, Jiachen Wang, Dafang Zhong, Xiaofang Zhang, Jing Zhang

**Affiliations:** 1 Department of Medical Oncology, Fudan University Shanghai Cancer Center, Shanghai, China; 2 Department of Oncology, Shanghai Medical College, Fudan University, Shanghai, China; 3 Shanghai Institute of Materia Medica, Chinese Academy of Sciences, Shanghai, China; 4 Department of Biostatistics, School of Public Health, Fudan University, Shanghai, China; 5 Shanghai Allist Pharmaceuticals, Shanghai, China

**Keywords:** AST1306, ErbB family, Irreversible tyrosine kinase inhibitor, Phase I

## Abstract

**Background:**

AST1306 is an orally active irreversible small molecule inhibitor of EGFR (erbB1), HER2 (erbB2) and HER4 (erbB4) signaling. This is a phase I, open-label, dose-escalation study to evaluate the safety and tolerability, pharmacokinetics (PK), and preliminary anti-tumor effects of oral AST1306. In addition the effects of food on PK was tested.

**Methods:**

A modified Fibonacci 3 plus 3 dose-escalation design was employed to determine the dose-limiting toxicity (DLT) and recommended phase II dose (RP2D) in patients with advanced solid tumors. The following dose levels were investigated: once daily (QD) at two dose levels (400-and 800 mg), twice daily (BID) in five dose levels (600-, 800-, 1000-, 1200- and 1500 mg), and three times daily (TID) in three dose levels (800-, 1000- and 1200 mg). In the PK and extension study, at least eight patients per dose cohort in three dose levels (maximum tolerated dose [MTD], one or two doses level lower than the MTD) were enrolled to evaluate the PK profiles.

**Results:**

Seventy-one patients were enrolled, with breast (n = 22) and lung cancers (n = 14) being the most common primary cancers. The most frequent drug-related adverse events were grade 1 to 3 diarrhea and rash, grade 1 to 2 fatigue. During dose escalation, the key DLT was grade 3 diarrhea observed in 5 patients at 1000 mg BID (n = 1), 1500 mg BID (n = 1), 800 mg TID (n = 1) and 1200 mg TID (n = 2). AST1306 was rapidly absorbed and had moderate to high clearance. PK concentration parameters increased with dose over the range evaluated, with no evidence of accumulation over time. Under fed conditions, the mean T_max_ was prolonged, C_max_ was increased, and AUC_0-∞_ was raised. Of the 55 evaluable patients, 7 patients experienced partial responses, including 5 with breast cancer, 1 with lung cancer, and 1 with gastric cancer. The best response with stable disease for ≥ 6 months was achieved in 7 patients.

**Conclusions:**

Based on the DLT and PK profile, the RP2D was defined as 1000 mg TID with evidence of preliminary anti-tumor activity. Further studies are recommended.

## Introduction

The epidermal growth factor (erbB) family of membrane receptor tyrosine kinases consists of the epidermal growth factor receptor (EGFR; erbB1) and human epidermal growth factor receptor (HER) 2 (erbB2), HER3 (erbB3) and HER4 (erbB4) [1]. The roles of the erbB family members are well documented in many types of cancer. A number of small-molecule tyrosine kinase inhibitors (TKIs) (e.g., gefitinib, erlotinib, lapatinib) and antibodies (eg, trastuzumab, cetuximab, panitumumab) targeting the erbB family have been developed to treat breast, colorectal, lung, gastric and head and neck squamous cell cancers [2]. Despite the improvements achieved in patients treated with reversible inhibitors of EGFR or EGFR and HER2, primary and acquired resistances to these agents remain a clinical challenge. This is exemplified by the development of resistance to EGFR tyrosine kinase inhibitor (TKI), through emergence of a secondary T790M EGFR mutation in non-small-cell lung carcinoma (NSCLC) [3], which occurs in 50% of the patients with lung adenocarcinoma [4]. Irreversible erbB family blocker may be beneficial to the patients with EGFR-TKI resistant NSCLC and trastuzumab resistant HER2-positive metastatic breast cancer (MBC) [5].

AST1306 (Shanghai Allist Pharmaceuticals, China) is an orally active, highly selective irreversible inhibitor of the erbB family receptor tyrosine kinases (HER1, HER2, and HER4) [6]. In preclinical trials, the IC_50_ values of AST1306 inhibiting EGFR and HER2 were 0.5 and 3 nmol/L, respectively, which were 5-15-fold more potent than afatinib and dacomitinib. AST1306 could potently inhibit the EGFR T790M mutant, exhibiting an IC_50_ value of 12 ± 2 nmol/L, which is similar to afatinib (10 nmol/L) and approximately 500-fold more potent than lapatinib [6-10]. In human tumor xenograft models that expressed or overexpressed HER family members, AST1306 showed antitumor activities, especially against those with HER2 overexpression or EGFR T790M mutant tumors [6].

The primary aims of this phase I, open-label, dose-escalation study (http://www.chictr.org/cn/identifier:ChiCTR-ONC-10000893) are to assess the safety and tolerability of AST1306 by dose-limiting toxicity (DLT) and maximum tolerated dose (MTD), determine the clinically recommended dose and schedule, characterize the pharmacokinetic (PK) profile of the drug following single and multiple dosing, and investigate the effect of food on the PK of this oral agent in Chinese patients with advanced solid tumors. The secondary endpoint was to assess antitumor activity.

## Materials and methods

### Patient population

Eligible patients were aged 18–75 years with histologically or cytopathologically confirmed advanced solid tumors. Patients had to either be refractory to, or intolerant of standard treatment known to provide clinical benefit for their metastatic cancers, or to be unable to afford them. The other inclusion criteria were as follows: Eastern Cooperative Oncology Group (ECOG) performance status of 0–2; adequate hematology (absolute neutrophil count ≥ 1.5 × 10^9^/L, platelet count ≥ 80 × 10^9^/L, hemoglobin ≥ 9 g/dL); normal liver function (serum bilirubin ≤ 1.5 × upper limit of normal [ULN]; aspartate aminotransferase and alanine aminotransferase ≤ 2.5 × ULN, except in patients with liver metastases) and kidney function (serum creatinine ≤ 1.5 × ULN); at least one measurable disease according to the Response Evaluation Criteria in Solid Tumors (RECIST) 1.0; life expectancy of ≥ 3 months; and no anticancer treatment for at least 4 weeks prior to enrollment in the study.

Exclusion criteria included the following: pregnant or breast feeding patients, any clinically significant gastrointestinal abnormalities that influence oral administration, uncontrolled or significant cardiovascular disease, left ventricular ejection fraction (LVEF) < 40%, metastases to the central nervous system (symptomatic and/or requiring treatment), active serious infection, and bleeding diathesis or coagulopathy.

### Study design and treatment

This was the first-in-human phase I dose-escalation study of AST1306 for patients with advanced solid tumors. AST1306 was orally administered without food for one day followed by two days off to evaluate single dose PK profiles. After that, it was continuously administered for 21 days and two days off to assess multiple dose PK profiles and safety. Once daily in two dose levels (400 mg QD and 800 mg QD) was first explored and according to the PK profile, with shorter t_1/2_ of 3–5 hours, twice daily in five dose levels (600 mg BID, 800 mg BID, 1000 mg BID, 1200 mg BID and 1500 mg BID) were then investigated. Three times daily in three dose levels (800 mg TID, 1000 mg TID and 1200 mg TID) were explored subsequently. Treatment was then repeated at the same dose level in 28-day cycles until unacceptable toxicities, disease progression or the patient’s withdrew from the study. PK extension study was performed to enroll additional patients up to at least eight patients per dose cohort in three dose levels (MTD, one or two doses level lower than the MTD) for further evaluation of PK profiles.

For the food-effect study, an open, two-way crossover design was adopted to assess the effect of food on the oral bioavailability of AST1306 at the MTD dose level (Additional file 1: Figure S1). On day 1, 6 subjects were fasted and 6 were fed, and all 12 subjects received a single dose of AST1306. Following a 2-day washout period, subjects from the fasted group were fed and vice versa. On “fasting” days (day 1 or 4), subjects were required to fast for a minimum of 2 h prior to dosing, and for at least 1 h post-dosing. On “fed” days (day 1 or 4), subjects were fasted overnight and were required to ingest a standard high-fat breakfast within 30 min prior to dosing. PK sampling then took place on day 1 and day 4. The PK sampling time points were pre-dose, 0.5, 1, 1.5, 2, 2.5, 3, 4, 6, 8, 12, and 24 hours after dosing.

This study was conducted in compliance with the ethical principles derived from the Declaration of Helsinki and all the International Conference on Harmonization (ICH) Good Clinical Practice (GCP) guidelines. The protocol, all the amendments and informed consent documentations were reviewed and approved by the Institutional Review Board at Fudan University Shanghai Cancer Center. All patients provided written, signed informed consent prior to entry into the trial.

### Dose escalation and safety assessment

Safety and tolerability were evaluated and assessed by investigators throughout the study and up to 14 days after the final dose of AST1306 according to Common Terminology Criteria for Adverse Events (CTC-AE) version 4.03. Laboratory evaluations and electrocardiogram were conducted at screening; every week of first and second cycles; every two weeks of third cycle and every four weeks of subsequent cycles. AEs assessment, vital signs, physical examinations and echocardiography were performed.

A modified Fibonacci 3 plus 3 dose-escalation design was employed. Tolerability was evaluated according to the type and frequency of DLTs observed from single-day-dose administration until day 21 of the first cycle of continuous dosing. DLTs were defined as follows: grade ≥ 3 non-hematologic toxicity (except nausea, vomiting and diarrhea); grade ≥ 3 nausea, vomiting or diarrhea uncontrollable with standard supportive care; grade 4 neutropenia or febrile neutropenia; and grade 4 thrombocytopenia or bleeding requiring a platelet transfusion. If two or more patients experienced DLTs in three to six patients for each cohort, dose escalation was terminated and the dose level below was defined as the MTD.

Dose interruptions were permitted for no more than 14 days for patients experiencing DLTs in the first cycle or intolerable toxicities in the subsequent cycles, with treatment resumed at the next lower dose level upon adequate recovery (grade ≤ 1). More than two dose level reductions were not allowed.

Patients in the PK extension study and food-effect study were also included to evaluate safety further.

### Evaluation of antitumor activity

Antitumor activity was assessed by tumor measurements according to RECIST 1.0 and performed at 8-week intervals. The primary efficacy endpoint was progression free survival (PFS), which was defined as the time from the first administration until disease progression or death. Overall survival (OS) and response rate were also assessed. Clinical benefit rate was measured and defined as the proportion of patients with complete response, partial response and stable disease (SD) ≥ 6 months.

### Pharmacokinetic analysis

All PK sampling was performed in the first cycle. Plasma concentrations of AST1306 single-day dosing were determined at the following distinct time points: pre-dose, 0.5 h, 1 h, 1.5 h, 2 h, 3 h, 4 h, 6 h, 8 h, 10 h, 12 h, 24 h, 36 h, and 48 h after the single dose administration in the 400 mg QD and 800 mg QD dose cohorts. For the next five dose cohorts with twice daily (once every 12 hours) administration, the time points of plasma sample collection were as follows: pre-dose, 0.5 h, 1 h, 1.5 h, 2 h, 3 h, 4 h, 6 h, 8 h, and 12 h after the first dose administration, and pre-dose, 1 h, 2 h, 3 h, 4 h, 6 h, 8 h, 12 h, and 24 h after the second dose administration. Three dose cohorts with three times daily (once every 8 hours) administration were further investigated with distinct time points for plasma collecting (pre-dose, 0.5 h, 1 h, 1.5 h, 2 h, 3 h, 4 h, 6 h, and 8 h after the first dose administration; 1 h, 2 h, 3 h, 4 h, 5 h, 6 h, and 8 h after the second dose administration; 1 h, 2 h, 3 h, 4 h, 8 h, 12 h, 24 h, and 36 h after the third dose administration). PK parameters of AST1306 were also determined on days 10, 23 (pre-dose and 2 h after the first dosing), and 24 (steady state, the same time points as the first-day dosing) of continuous treatment. The PK sampling schedule for the PK extension phase was totally the same as that for the dose escalation study.

PK analysis was conducted using Phoenix WinNonlin® software (version 6.1, Pharsight, Mountainview, CA). Standard non-compartmental methods were used to calculate the area under the plasma concentration-time curve over the time interval from 0 to 24 h (AUC_0–24,ss_), maximum measured concentration of the analyte in plasma (C_max_), and terminal half-life of the analyte in plasma (t_1/2_). Time from dosing to the maximum concentration of the analyte in plasma (t_max,_) was reported as a median value. The concentration at the trough level was determined using the concentration at 24 h (C_24_) on steady state days.

The power model method was used to assess the dose proportionality. The differences between fed and fasting conditions were assessed by ANOVA, and 90% confidence intervals for ratios were given.

## Results

### Patient characteristics

From May 2010 to April 2012, a total of 71 patients with advanced solid tumors were recruited into this study. Twelve patients were enrolled in the food-effect study at the MTD dose level. The baseline demographic characteristics of these patients are provided in Table 1. Patients had a variety of cancer types, with the most common being breast cancer and NSCLC. Patients were heavily pretreated, and 84.5% of them had received ≥ 3 lines of therapy regimens. All patients were included in safety and PK evaluation. Four patients who had no measurable lesions, and 12 patients who experienced DLTs or withdrew their informed consents before the first time point of tumor measurement, were not included in tumor response evaluation. Thus, 55 patients were evaluable for efficacy assessment. Fifteen of 17 evaluable breast cancer patients were HER2 positive (Immunohistochemistry+++ and/or FISH/CISH+), and of these, 7 patients had received trastuzumab and 4 patients had received lapatinib. Among the 12 evaluable NSCLC patients, 6 patients harbored activating EGFR mutations, and 8 patients had a treatment history of reversible EGFR TKIs. The median duration of time on study for all patients was 3.2 months (range 0.2-16.8 months).

**Table 1:** Demographics of All Treated Patients (n = 71)

**Demographic**	**No. of patients**	**%**
Gender		
Male	30	42.3
Female	41	57.7
Median age, years	56	
Range	28-75	
Cancer type		
Breast	22	31.0
Lung	14	19.7
Rectum	9	12.7
Colon	7	9.9
Others	5	7.0
Ovary	4	5.6
Nasopharynx	3	4.2
Stomach	5	7.0
Cervix	1	1.4
Unknown primary	1	1.4
ECOG performance status		
0	17	23.9
1	54	76.1
2	0	0
Prior treatment		
Chemotherapy	71	100.0
1 regimen	5	7.0
2 regimens	6	8.5
≥3 regimens	60	84.5
Surgery	56	78.9
Radiotherapy	36	50.7
Hormone therapy	11	15.5
Targeted therapy	32	45.1
Others	21	29.6

*Abbreviation*: *No*. number, *ECOG* Eastern Cooperative Oncology Group.

### Assessment of DLT and MTD

In total, five patients developed DLTs during the dose escalation study, one patient each from the 1000 mg BID, 1500 mg BID, and 800 mg TID cohorts, and two patients from the 1200 mg TID cohort. There were no DLTs with QD dosing. All DLTs were grade 3 diarrhea which was observed from single-day-dose administration until day 21 of the first cycle of continuous dosing and was not ameliorated with appropriate intervention. Based on the DLT events mentioned above and PK results listed below, the MTD and recommended phase II dose (RP2D) for AST1306 was defined at 1000 mg TID when administered in a continuous-dosing schedule. PK extension study was performed at MTD dose (1000 mg TID, n = 3) and one or two doses level lower than the MTD (800 mg TID, n = 5; 600 mg TID, n = 9). In addition, one further case of grade 3 diarrhea was observed at 800 mg TID in the PK extension phase but not considered in dose escalation decision.

### Safety and tolerability

All enrolled patients were included in the safety analysis. Overall, AST1306 was well-tolerated, with mainly grade 1 to 2 AEs, and no observed grade 4 to 5 AEs. Sixty-eight patients experienced AEs that were considered to be study drug-related (Table 2). Diarrhea (n = 61, 85.9%), fatigue (14, 19.7%) and rash (12, 16.9%) were the most common treatment-related AEs and usually occurred within the first 2 weeks of treatment. Diarrhea was managed effectively with loperamide or temporary interruption of AST1306. Rash was well controlled in most patients with topical antibiotics (mainly tetracycline) and corticosteroids or interruption of AST1306.

**Table 2:** Treatment-related AEs

**AEs**				**Diarrhea**		**Fatigue**		**Rash**		**Vomiting**		**Proteinuria**		**ALT increased**		**Anorexia**		**Hand-foot syndrome**	
Grade				1-2	3	1-2	3	1-2	3	1-2	3	1-2	3	1-2	3	1-2	3	1-2	3
Dose Cohort	QD	400mg (n = 1)	Couse 1	-	-	-	-	1	0	-	-	-	-	-	-	-	-	-	-
			All Course	-	-	-	-	1	0	-	-	-	-	-	-	-	-	-	-
		800mg (n = 3)	Couse 1	-	-	-	-	1	0	-	-	-	-	-	-	-	-	-	-
			All Courses	-	-	-	-	1	0	2	0	-	-	-	-	-	-	-	-
	BID	600mg(n = 3)	Couse 1	1	0	-	-	-	-	-	-	-	-	-	-	-	-	1	0
			All Courses	1	0	-	-	1	0	-	-	-	-	-	-	-	-	1	0
		800mg (n = 3)	Couse 1	1	0	-	-	1	1	-	-	-	-	-	-	-	-	-	-
			All Courses	1	1	-	-	1	0	-	-	-	-	1	0	-	-	-	-
		1000mg (n = 6)	Couse 1	1	4	-	-	1	0	1	0	-	-	-	-	-	-	-	-
			All Courses	1	5	-	-	1	0	1	0	-	-	-	-	-	-	-	-
		1200mg (n = 3)	Couse 1	2	0	-	-	-	-	-	-	-	-	-	-	-	-	-	-
			All Courses	2	0	3	0	-	-	-	-	-	-	-	-	-	-	-	-
		1500mg (n = 6)	Course 1	4	2	-	-	1	0	-	-	-	-	-	-	-	-	-	-
			All Courses	4	3	-	-	1	0	-	-	-	-	-	-	-	-	-	-
	TID	600mg (n = 0 + 9)*	Couse 1	4	2	-	-	-	-	1	1	-	-	-	-	-	-	-	-
			All Courses	5	2	2	0	-	-	3	0	2	0	-	-	1	0	-	-
		800mg(n = 8 + 5)*	Couse 1	5 + 3	3 + 1	-	-	0 + 0	0 + 0	-	-	-	-	-	-	-	-	-	-
			All Course	5 + 4	4 + 2	1 + 0	0 + 0	1 + 0	0 + 0	-	-	0 + 3	0 + 0	2 + 0	0 + 1	1 + 1	0 + 0	1 + 0	0 + 0
		1000mg (n = 6 + 3 + 12)^†^	Couse 1	5 + 2 + 0	1 + 1 + 0	1 + 0 + 0	0 + 0 + 0	1 + 1 + 0	0 + 0 + 0	0 + 1 + 0	0 + 0 + 0	-	-	-	-	-	-	3 + 0 + 0	0 + 0 + 0
			All Courses	5 + 2 + 8	1 + 1 + 1	2 + 0 + 4	0 + 0 + 0	1 + 1 + 2	1 + 0 + 0	0 + 1 + 2	0 + 0 + 0	2 + 0 + 3	0 + 0 + 0	0 + 1 + 3	0 + 0 + 1	0 + 0 + 6	0 + 0 + 0	3 + 0 + 3	0 + 0 + 0
		1200mg (n = 3)	Couse 1	1	2	1	0	-	-	2	0	-	-	-	-	-	-	1	0
			All Courses	0	3	2	0	-	-	2	0	-	-	-	-	-	-	1	0
		Total	Couse 1	28	16	0	0	7	0	5	0	0	0	0	0	0	0	5	0
				39.4%	22.5%	0%	0%	9.9%	0%	7.0%	0%	0%	0%	0%	0%	0%	0	7.0%	0%
			All Courses	38	23	14	0	11	1	11	0	10	0	7	2	9	0	9	0
				53.5%	32.4%	19.7%	0%	15.5%	1.4%	15.5%	0%	14.1%	0%	9.9%	2.8%	12.7%	0%	12.7%	0%

*Dose escalation study + Pharmacokinetics extension study.

^†^Dose escalation study + Pharmacokinetics extension study + Food effect study.

*Abbreviations*: *AEs*. Adverse events.

Grade 3 study drug-related AEs occurred in 28 (39.4%) patients. The most common grade 3 drug-related AE was diarrhea (23, 32.4%). Other grade 3 drug-related AEs presented in lower frequencies and consisted of elevated ALT or AST (3, 4.2%), rash, anemia, hypokalemia and abdominal pain (1, 1.4% each). Five patients with DLT recovered from grade 3 diarrhea after dose interruption, dose reduction and appropriate medications. The main causes of discontinuation from treatment due to drug-related AEs were diarrhea (5 patients) and hypokalemia (1 patient). Seven patients (1 at 800 mg BID, 2 at 1000 mg BID, 2 at 1500 mg BID, 1 at 600 mg TID, 1 at 800 mg TID) had dose reductions and all due to diarrhea, in which 1 patient at 1000 mg BID experienced 2 dose reductions and the rest required 1 dose reduction. There was no significant decline in LVEF.

Serious AEs (SAE) occurred in 8 patients. Three of the 8 patients had SAE reported during the first cycle of treatment. In these 3 patients, one had grade 3 diarrhea and dehydration (1500 mg BID cohort), which was thought to be due to AST1306; one patient developed a lung infection and the last patient had increased pleural effusions and shortness of breath and both were considered to be unrelated to AST1306. The other five patients experienced SAEs after the first cycle. One of these patients was diagnosed with grade 3 AST1306-related diarrhea. The rest of the SAEs included 1 case of hyperbilirubinemia and 3 deaths within 14 days after the final dose of AST1306 due to disease progression.

### Pharmacokinetics

Additional patients were enrolled up to at least eight patients per dose cohort in three dose levels (600 mg TID, 800 mg TID and 1000 mg TID) in the PK extension study and food-effect study to evaluate PK profiles. Collected plasma samples were analyzed by dose cohort. The PK analysis population (n = 61) and the geometric mean PK parameters of AST1306 for single-day dosing and multiple dosing are summarized in Additional file 2: Table S1.

For single dose evaluation, C_max_ was achieved in 1.83 to 3.67 hours after oral administration. The plasma levels of AST1306 increased with increasing doses and varied considerably between patients. C_max_ and AUC_0–24 h_ showed a dose-dependent increase. The terminal half-life of AST1306 ranged between 3.48 to 5.56 hours on day 1.

For multiple-dose evaluation, C_max_ and AUC_0–24h_ showed a dose-dependent increase at dose levels of 400 mg QD to 1000 mg TID. Whereas, C_max_ and AUC_0–24h_ increased by a smaller amount at the 1200 mg TID dose level, and showed moderate-to-high inter-individual variability. The gMean terminal half-life ranged from 3.33 to 7.57 h on days 23–24. Multiple-dose exposure was no more than 3-fold greater than single-dose exposure across the entire dose range, as assessed by the mean accumulation ratio (R, AUCss on study day 23–24 to AUC_0-24h_ on study day 1; Additional file 2: Table S1). These results suggested that there was no major accumulation of AST1306 after repeated daily administration to patients.

With the same total daily dose, for example 1500 mg BID vs. 1000 mg TID, the C_avg_, AUC_0-24h_, AUC_0-t_ and AUC_0-∞_ of TID administration schedule were higher than those of QD and BID schedules. In addition, the fluctuation index (FI) of the TID schedule (137-204%) was lower than that of the BID (201-274%) or QD schedules (499-535%).

In the food-effect study, with a fed-fasted two-way crossover design, a total of 12 patients (6 patients each group) were enrolled to assess the initial effect of food on the oral bioavailability of AST1306 (Additional file 1: Figure S1). High-fat food intake before a single oral AST1306 dose of 1000 mg significantly altered and increased drug exposure (Additional file 3: Table S2). Under fed conditions, mean T_max_ was prolonged (4.25 hours fed; 2.38 hours fasted; p = 0.012), C_max_ was increased (139 ng/mL fed; 70.3 ng/mL fasted), and AUC_0-∞_ was raised (814 h*ng/mL fed; 331 h*ng/mL fasted).

### Antitumor activity

**Figure 1:**
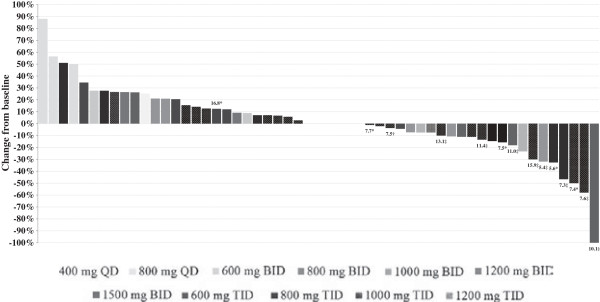
**Waterfall plot of best responses, as percentage decrease in tumor size by RECIST, of the target lesions in patients.** Fifty-five patients had measurable disease by RECIST and had at least 1 evaluation. The numbers above or below the bars represent the number of months the responders and patients with SD lasting ≥ 6 months received AST1306. * No EGFR or HER2 status; † EGFR exon 19/exon 20 double mutation; ‡ HER2 positive.

**Figure 2:**
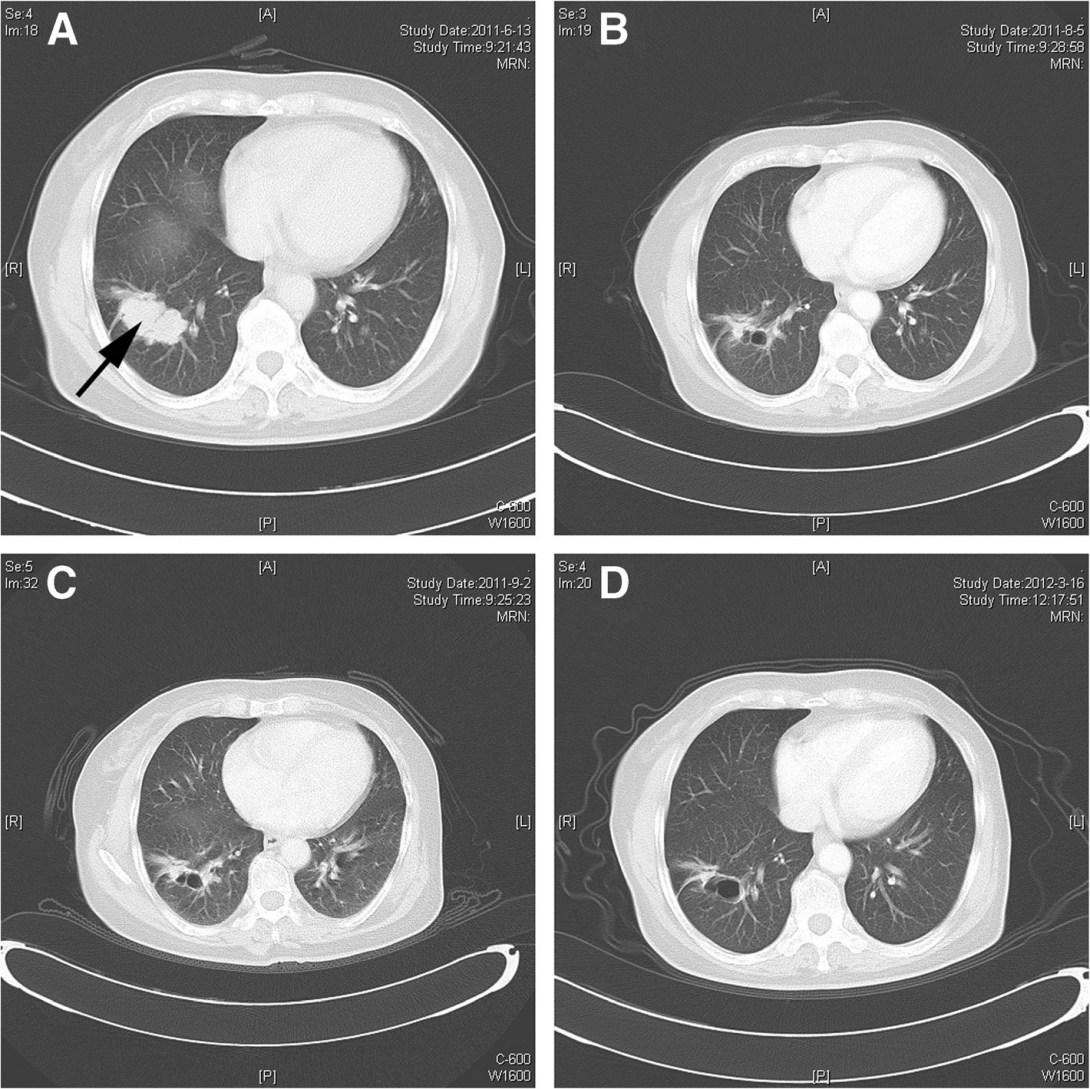
**A 67-year old female patient diagnosed with MBC who had experienced treatment failure after 2 chemotheraphy and 2 hormonal regimens, but did not receive trastuzumab.** She had confirmed PR at the 1500 mg BID dose level of AST1306, but withdrew from the study after 9 months due to continuous grade 2 diarrhea. **A**) CT imaging of the metastatic lesion prior to starting therapy, and **B**) shows the response to AST1306 two cycles after starting therapy. **C** and **D** shows continued response three and ten cycles after starting therapy.

Fifty five of the 71 patients were evaluable for efficacy. The waterfall plot of best responses in 55 evaluable patients was shown in Figure 1. Of the evaluable patients, 7 patients (5 with breast cancer, 1 with NSCLC, and 1 with gastric cancer) had confirmed PRs. Five of the 17 evaluable breast cancer patients achieved a PR and all responders were HER2 positive; 1 patient had been previously treated with trastuzumab and lapatinib (Figure 2). Among the 12 evaluable NSCLC patients, 1 patient had PR. This patient had failed 3 previous lines of chemotherapy, but was of unknown EGFR mutational status; in addition this patient had not previously received therapy with EGFR inhibitors. One PR was observed in a gastric adenocarcinoma patient who failed 2 lines of chemotherapy and 1 VEGFR2 TKI; however, the tumor tissue from biopsy was not enough to assess EGFR and HER2 status.

There were 7 patients who had SD for ≥ 6 months. Among those patients, 3 were breast cancer patients, all with HER2 positive; others were NSCLC (1), nasopharyngeal cancer (1), gastric cancer (1), and cervical cancer (1). The NSCLC patient achieving SD for ≥ 6 months had an EGFR sensitive mutation (exon 19 deletion) and previously responded to gefitinib. A secondary mutation of T790M in EGFR exon 20 was confirmed after progression on 15-month gefitinib treatment with subsequent lung wedge resection. The EGFR and HER2 status was not detected for the rest 3 patient (1 nasopharyngeal, 1 gastric, 1 cervical cancer).

As of the last updated data from July 25, 2013, 29 patients were still alive. The median follow-up time was 22.7 months. For 17 breast cancer patients, the median PFS and OS reached 5.5 (95% CI, 3.2–7.9) and 17.7 (95% CI, 16.1–19.3) months, respectively. For 12 NSCLC patients, the median PFS was 3.6 months (95% CI, 3.3–3.9) months and the median OS has not yet been reached.

## Discussion

We described a phase I trial of AST1306, which is an irreversible inhibitor of EGFR, HER2, and HER4. This study indicated that AST1306 was generally safe and well tolerated on a continuous dose schedule in patients with advanced solid tumors. Based on two DLTs in the 1200 mg TID cohort, 1000 mg TID was declared as the MTD for this study. Grade 3 diarrhea was the key DLT observed in the dose escalation study. Additionally, 3 patients in the PK extension study and 12 patients in food-effect study were treated with 1000 mg TID, and no grade ≥ 3 toxicities were found, indicating that 1000 mg TID was well tolerated. There were no unexpected toxicities observed in this study and safety profiles were similar to the agents that target erbB signaling [11-14]. Diarrhea was manageable by appropriate medications and dose interruption or reduction in this study. Five (7%) patients had to discontinue therapy because of diarrhea.

Besides diarrhea, grade 1–2 fatigue and grade 1–3 rash were also the most frequently reported AEs, which is similar to the other three irreversible erbB family blockers afatinib [15,16], dacomitinib [17] and neratinib [18,19]. The incidence of skin rashes caused by AST1306 (16.9% for all grades) was comparable to neratinib [18], however, it was much lower than afatinib [15,16] and dacomitinib [17]. The rash could be effectively managed using topical antibiotics and corticosteroids with no need for dose interruption or reduction.

Although in the dose escalation study, the PK information of several patients in multiple dose levels had been collected, it was not enough to fully evaluate the PK profiles. According to the regulations and guideline of phase I study in China [20], PK extension study was performed to enroll additional patients up to at least eight patients per dose cohort in three dose levels (MTD, one or two doses level lower than the MTD). PK analysis suggested a dose-proportional relationship over the dose range tested. All PK parameters displayed moderate to high variability within the expected range for orally administered TKIs (eg, lapatinib [21], erlotinib [22], gefitinib [23], and afatinib [24]). The terminal elimination half-life of AST1306 was relatively short and suitable for TID dosing. Compared with other TKIs (eg, lapatinib [21]), AST1306 has a lower bioavailability, which may be due to its poor aqueous solubility. Food significantly increased the plasma exposure of AST1306 and reduced its inter-individual variability, indicating that poor bioavailability could be improved by dosing with meals to achieve therapeutic concentrations. However, the differences in eating habits between patients and within a patient from one day to the next do exist. Under these circumstances, we suggest that the potential risks of toxicity or loss of efficacy (secondary to loss of appetite) currently preclude the routine use of food to reliably and consistently improve AST1306 bioavailability for chronic therapeutic use. AST1306 is recommended to take without food in the subsequent phase II study.

AST1306 showed promising anti-cancer activity in heavily pretreated advanced solid tumor patients; seven (12.7%) patients demonstrated a confirmed PR and seven (12.7%) patients demonstrated SD ≥ 6 months. Twenty nine percent (5 of 17) of breast cancer patients achieved PR: all responders were HER2 positive, 1 of those had previous trastuzumab and lapatinib exposure. SD ≥ 6 months was observed in 3 breast cancer patients. As an irreversible HER2 inhibitor, consistent with its preclinical data, AST1306 not only had benefit to HER2 inhibitor naive breast cancer patients, but also showed potential benefit to HER2 inhibitor pretreated patients.

Partial response was reported in one NSCLC patient with unknown EGFR status. One NSCLC patient with SD lasting ≥ 6 months, and who had a mutation in EGFR had progressed after 15-month gefitinib treatment; he then subsequently developed a T790M mutation which was defined prior to entry to this study. The emergence of a T790M missense mutation is commonly associated with acquired resistance to first-generation EGFR inhibitors in NSCLC [8], and is detected in a subpopulation of cells in some tumors even before treatment with an EGFR inhibitor [25]. The ability of AST1306 to inhibit the growth of cells exhibiting the T790M mutant EGFR indicates that this agent also deserves further evaluation in NSCLC patients that are naïve and resistant to EGFR inhibitor. Interestingly, a PR was observed in a gastric adenocarcinoma patient with unknown EGFR and HER2 status. EGFR and HER2 are overexpressed in 15-45% of patients with gastric or gastro-esophageal junction cancer, thereby making them potential targets [26]. Although two phase III trials (LOGiC [27] and TyTAN [28]) for lapatinib, which is an EGFR and HER2 kinase inhibitor, failed to reach the primary endpoint, lapatinib did show modest single-agent activity in advanced/metastatic gastric cancer patients with a response rate of 9% [26].

According to the preliminary efficacy of AST1306, HER2 positive breast cancer and EGFR mutant NSCLC are the two cancer types recommended to be further studied in the phase II study. Also, the much lower incidence of skin rashes than afatinib [15,16] and dacomitinib [17] makes AST1306 worthy of being further investigated.

In conclusion, oral AST1306 is well-tolerated when administered continuously, with promising antitumor activity in multiple tumor types. The MTD of AST1306 was determined to be 1000 mg TID. This dose is the recommended dose of AST1306 for subsequent phase II clinical trials.

## Abbreviations

PK: Pharmacokinetic; DLT: Dose-limiting toxicity; RP2D: Recommended phase II dose; MTD: Maximum tolerated dose; EGFR: Epidermal growth factor receptor; TKI: Tyrosine kinase inhibitor; NSCLC: Non-small-cell lung carcinoma; MBC: Metastatic breast cancer; ECOG: Eastern Cooperative Oncology Group; RECIST: Response evaluation criteria in solid tumors; LVEF: Left ventricular ejection fraction; ICH: International Conference on Harmonization; GCP: Good clinical practice; CTC-AE: Common terminology criteria for adverse events; PFS: Progression free survival; OS: Overall survival; SD: Stable disease; SAE: Serious adverse events; FI: Fluctuation index.

## Competing interests

The authors declared that they have no competing interests.

## Authors’ contributions

Conceived and designed the study: JNC, JL. Performed the study: JZ, JNC, JL, ZYC, WP, SS, JCW. Analyzed the data: JZ, JNC, YFZ, NQZ, DFZ, XFZ, JZ. Wrote the paper: JZ, JNC, JL. All authors have read and approved the final manuscript.

## Supplementary Material

Additional file 1: Figure S1 Randomization to one of two treatment sequences each comprising two treatment periods (fasted and fed). Click here for file

Additional file 2: Table S1 Geometric Mean and CV Pharmacokinetic Parameters of AST1306 on Days 1 and 23-24 (ss) After Single and Multiple Oral Administration. Click here for file

Additional file 3: Table S2 Pharmacokinetic parameters (range) for subjects receiving AST1306 1000 mg with and without food. Click here for file
