# Supplementary figures and images for: A phase I study of AST1306, a novel irreversible EGFR and HER2 kinase inhibitor, in patients with advanced solid tumors
Source: J Hematol Oncol. 2014 Mar 11;7:22. doi: 10.1186/1756-8722-7-22 (PMC4007625; doi:10.1186/1756-8722-7-22)

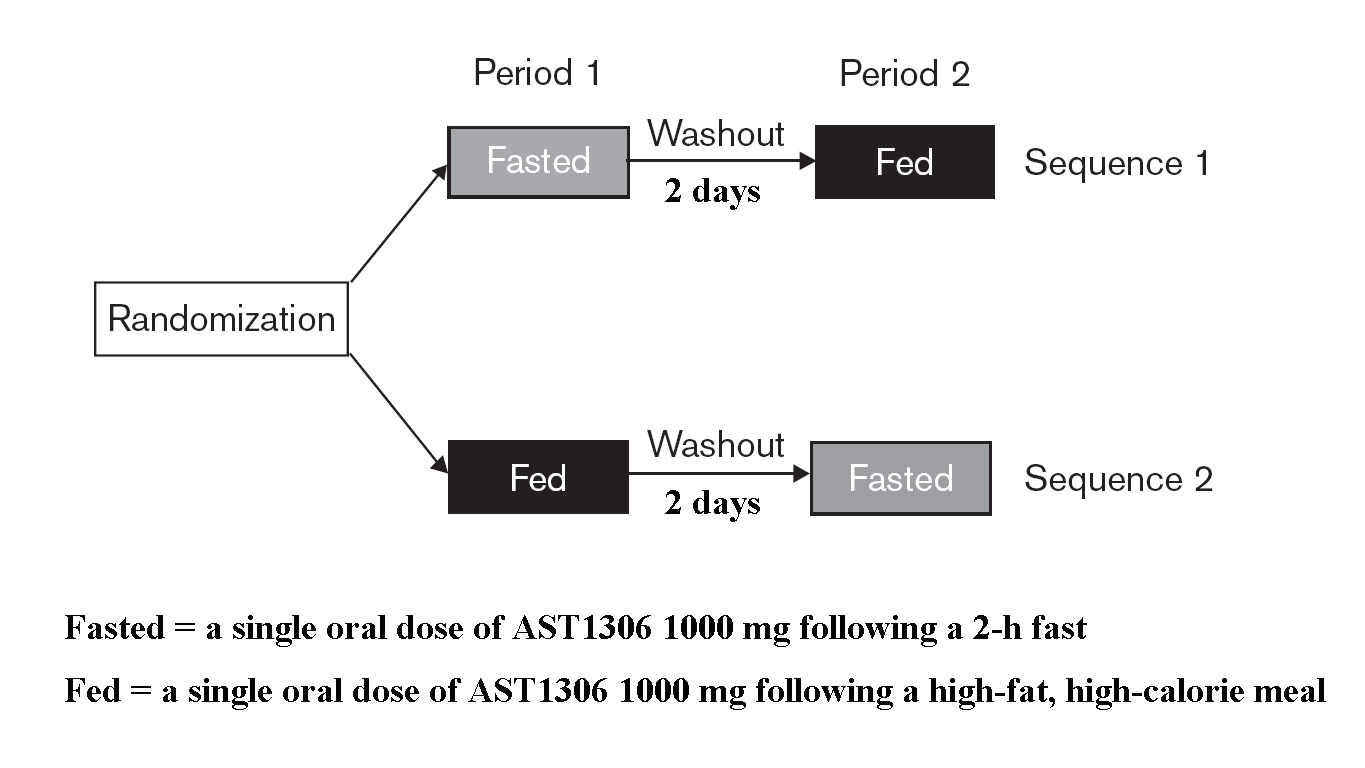

Supplement: Additional file 1: Figure S1 — Randomization to one of two treatment sequences each comprising two treatment periods (fasted and fed). [file 1756-8722-7-22-S1.tiff]
