# Supplementary material for: A phase I study of AST1306, a novel irreversible EGFR and HER2 kinase inhibitor, in patients with advanced solid tumors
Source: J Hematol Oncol. 2014 Mar 11;7:22. doi: 10.1186/1756-8722-7-22 (PMC4007625; doi:10.1186/1756-8722-7-22)
Supplement: Additional file 3: Table S2 — Pharmacokinetic parameters (range) for subjects receiving AST1306 1000 mg with and without food. [file 1756-8722-7-22-S3.docx]

**Supplementary Table 2.** Pharmacokinetic parameters (range) for subjects receiving AST1306 1000 mg with and without food

|  | C_max_ (ng/mL) | AUC_0–t_ (h*ng/mL) | AUC_0-∞_ (h*ng/mL) | T_max_ (h) | t_1/2z_ (h) | CLss/F (L/h) |
| --- | --- | --- | --- | --- | --- | --- |
| Fasted^a^ (n =12) | 70.3 (17.1-151.0) | 311 (102-713) | 331 (113-727) | 2.38 (1.50-4.00) | 6.99 (2.41-19.90) | 3970 (1370-8890) |
| Fed^a^ (n =12) | 139.0 (59.4-218.0) | 797 (413-1600) | 814 (427-1640) | 4.25 (2.50-8.00) | 4.19 (2.87-6.80) | 1360 (608-2340) |
| Treatment ratio^b^ | 228 (170-305) | 284 (219-368) | 269 (208-348) |  |  |  |

a Geometric mean values (ranges) presented.

b Ratios (%) of fed/fasted glsmeans (90% CIs) presented; except for Tmax, where medians (range) presented.
